# Auswirkungen thermischer Belastungen auf die Gesundheit – eine bundesweite Analyse auf Grundlage von GKV-Routinedaten zwischen 2012–2021

**DOI:** 10.1007/s00103-024-03968-5

**Published:** 2024-10-24

**Authors:** Jobst Augustin, Sandra Hischke, Peter Hoffmann, Dante Castro, Nadia Obi, Alice Czerniejewski, Roman Dallner, Laurens M. Bouwer

**Affiliations:** 1 Institut für Versorgungsforschung in der Dermatologie und bei Pflegeberufen (IVDP), Universitätsklinikum Hamburg-Eppendorf (UKE), Hamburg, Deutschland; 2 Climate Service Center Germany (GERICS), Helmholtz-Zentrum Hereon, Hamburg, Deutschland; 3 Institut für Arbeitsmedizin und Maritime Medizin, Universitätsklinikum Hamburg-Eppendorf (UKE), Hamburg, Deutschland; 4 Institut für Medizinische Biometrie und Epidemiologie, Universitätsklinikum Hamburg-Eppendorf (UKE), Hamburg, Deutschland; 5 BKK-Landesverband NORDWEST KdöR, Essen, Deutschland

**Keywords:** Klimawandel, Hitze, Public Health, Morbidität, Deutschland, ICD-Diagnosen, Climate change, Heat, Public health, Morbidity, Germany, ICD diagnosis

## Abstract

**Hintergrund:**

Inwieweit sich mit GKV-Routinedaten Assoziationen von thermischer Belastung und hitzeassoziierten Erkrankungen abbilden lassen, ist unklar. Ziel dieser Untersuchung ist die Analyse des Zusammenhangs zwischen klimatischer Variabilität und hitzeassoziierten Erkrankungen auf Grundlage von Routinedaten.

**Methoden:**

Die Studie basiert auf Abrechnungsdaten (ambulant und stationär) der Betriebskrankenkassen der Jahre 2012–2021 und umfasst circa elf Millionen gesetzlich Krankenversicherte. Berücksichtigt wurden vier hitzeassoziierte ICD-10-Diagnosen: T67 (Schäden durch Hitze und Sonnenlicht), E86 (Volumenmangel), N17 (akutes Nierenversagen) und N19 (Niereninsuffizienz). Die thermischen Bedingungen wurden mittels meteorologischer Variablen quantifiziert. Die Auswertung erfolgte bundeslandspezifisch für die 2. und 3. Quartale (Q2, Q3) mittels deskriptiver Verfahren und Korrelationsanalysen mit Messwiederholungen.

**Ergebnisse:**

Die Jahre 2016, 2018 und 2020 sind mit hohen Temperaturen aufgefallen. Im Vergleich zu Q2 wurde in Q3 im Durchschnitt aller Jahre eine höhere thermische Belastung verzeichnet, einhergehend mit mehr hitzeassoziierten Diagnosen. So sind bundesweit die Diagnosen für T67 (ambulant) mit der Anzahl heißer Tage (r_mw_ = 0,86 (0,81; 0,90)) korreliert. Wenngleich die thermische Belastung in Q2 geringer ist, zeigt sich aber auch hier ein ähnlicher Zusammenhang (r_mw_ = 0,76 (0,68; 0,82)). Diese markante Assoziation blieb auch bei Betrachtung der Bundesländer erhalten.

**Diskussion:**

Der Beitrag zeigt, dass sich auch in GKV-Routinedaten Assoziationen zwischen thermischer Belastung und Morbiditätsmarkern finden lassen. Vor dem Hintergrund einer klimawandelbedingten Zunahme hoher thermischer Belastungen zeigt dieser Beitrag die Notwendigkeit von Anpassungsmaßnahmen.

## Abstract (en)

**Background:**

The extent to which statutory health insurance (SHI) data can be used to map associations between thermal stress and heat-related diseases is still unclear. The aim of this study is to analyse the association between climate variability and heat-associated diseases using insurance data.

**Methods:**

The study is based on claims data (outpatient and stationary) from health insurance companies in Germany for the years 2012–2021, covering approximately 11 million people with statutory health insurance. Four heat-related ICD-10 diagnoses were considered: T67 (heat and sun damage), E86 (volume depletion), N17 (acute renal failure) and N19 (renal insufficiency). Thermal conditions were quantified using meteorological variables. The evaluation was carried out on a federal state-specific basis for the second and third quarters (Q2, Q3) using descriptive methods and correlation analyses with repeated measurements.

**Results:**

The years 2016, 2018 and 2020 were characterised by high temperatures. Compared with Q2, Q3 was associated with higher thermal stress and more heat-related diagnoses on average during the entire observational period. Nationally, diagnoses of heat and sun damage (outpatient) correlated with the number of hot days (r_mw_ = 0.86 [0.81; 0.90]). Although heat stress is lower in the second quarter, a similar correlation is found here (r_mw_ = 0.76 [0.68; 0.82]). This striking association was also maintained when analysing the federal states specifically.

**Discussion:**

The article shows that associations between thermal stress and morbidity can also be found in routine SHI data. Against the background of an increase in high thermal stress due to climate change, this article highlights the need for adaptation measures.

## Einleitung

Die Beeinträchtigung der menschlichen Gesundheit aufgrund zunehmender thermischer Belastungen zählt vermutlich zu den bedeutendsten Auswirkungen des Klimawandels auf die Gesundheit. Wenngleich auch Kälte die Gesundheit schädigen kann, stehen hohe Lufttemperaturen bzw. Hitzeextreme und ihre Auswirkungen auf die Bevölkerung besonders im Fokus. Ursache dafür ist die markante Zunahme von Ereignissen hoher thermischer Belastung während der letzten 2 Jahrzehnte. So konnten Rousi et al. [1] Europa als Hitzewellen-Hotspot identifizieren, der in den letzten 42 Jahren hinsichtlich der Temperaturentwicklung einen 3‑ bis 4‑mal schnelleren Aufwärtstrend als der Rest der nördlichen Mittelbreiten aufweist. Das Jahr 2023 war das wärmste Jahr in Deutschland seit 1881, mit einem Mittelwert von +2,3 °C über dem vorindustriellen Niveau (1881–1910; [2]). Damit war es auch das 13. Jahr in Folge, welches über dem vieljährigen Mittelwert von 1961–1990 lag. Betrachtet man nur den Sommer und beispielsweise die Anzahl „heißer Tage“ (Tage mit einer Höchsttemperatur von 30 °C oder mehr), zeigen sich zwar jährlich Schwankungen, aber auch hier wird der Trend zur Erwärmung deutlich [3]. Die Jahre 2003 (19 Tage), 2015 (17,6 Tage), 2018 (20,4 Tage) und 2022 (17,3 Tage) waren, gemittelt über Gesamtdeutschland, die Jahre mit der höchsten Anzahl heißer Tage seit 1950 [4].

Die Folgen hoher thermischer Belastungen auf den Körper sind vielfältig. Die Weltgesundheitsorganisation (WHO; [5]) unterscheidet zwischen den direkten und indirekten Folgen starker Hitzebelastungen. Zu den direkten Folgen werden hitzebedingte Gesundheitsstörungen gezählt. Neben Hitzekrämpfen oder einem Hitzschlag ist hier der Volumenmangel (Internationale Klassifikation der Krankheiten: ICD-10-Code E86) besonders relevant. Darunter wird einerseits ein Flüssigkeitsdefizit, unter anderem aufgrund unzureichender Wasserzufuhr, verstanden (Dehydration), andererseits ist der Volumenmangel durch einen zusätzlichen Verlust von Natrium gekennzeichnet (Hydrovolämie) und kann durch Vorerkrankungen der Niere und Diuretika-Einnahme begünstigt werden. Darüber hinaus kann eine starke Hitzebelastung zu einer Verschlimmerung bestehender Erkrankungen (z. B. Atemwegserkrankungen, Diabetes mellitus, Nierenerkrankungen) und zu vorzeitigen Todesfällen aufgrund von Atemwegserkrankungen, Herz-Kreislauf-Erkrankungen oder sonstigen Erkrankungen führen [6, 7]. Zu den indirekten Folgen werden die Auswirkungen auf das Gesundheitswesen (z. B. mehr Rettungseinsätze), erhöhtes Unfallrisiko (z. B. Arbeitsunfälle), erhöhtes anderweitiges Risiko (z. B. durch Wasser übertragene Infektionserkrankungen) und Gefährdung der Infrastruktur (z. B. Wasserversorgung) gezählt. Darüber hinaus zeigt sich ein Zusammenhang zu psychischen Erkrankungen, wie ein mit steigenden Temperaturen erhöhtes Suizidrisiko oder aggressives Verhalten [8].

Die epidemiologische Evidenz des Zusammenhangs von Hitze und Mortalität wurde in zahlreichen Studien (z. B. [9–12]) untersucht, auch für Deutschland. Winklmayr et al. [12] haben beispielsweise mithilfe eines generalisierten additiven Modells auf Grundlage von wöchentlichen Daten zur Gesamtmortalität und zu mittleren Temperaturen im Zeitraum 1992–2021 die Anzahl hitzebedingter Sterbefälle analysiert. Es zeigte sich, dass die hohen Sommertemperaturen 2018–2020 in allen 3 Jahren zu einer statistisch signifikant höheren Anzahl von hitzebedingten Sterbefällen gegenüber den Vorjahren geführt haben. Im Jahr 2018 gab es in Deutschland etwa 8700 hitzebedingte Sterbefälle, in 2019 etwa 6900 und in 2020 etwa 3700. Im Zeitraum 2012–2021 lag die durchschnittliche Anzahl hitzebedingter Sterbefälle bei etwa 3500; in den historischen Hitzejahren 1994 und 2003 bei jeweils etwa 10.000 [12].

Aus Public-Health-Perspektive ist vor allem aber die Assoziation von Hitze mit Morbidität von Bedeutung. So zeigen beispielsweise Phung et al. [13], dass die Inzidenz von Erkrankungen des Herz-Kreislauf-Systems während einer Hitzeperiode um 2,2 % pro 1 °C Zunahme der Lufttemperatur ansteigt. Chen et al. [14] und Sun et al. [15] haben Zusammenhänge zwischen Herzinfarkten und hohen Temperaturen aufzeigen können. Nach Bunker et al. [16] sind Erkrankungen der Atemwege, nach den Herz-Kreislauf-Erkrankungen, die zweithäufigste Ursache für Morbidität im Rahmen von Hitzeperioden. Dies betrifft vor allem urbane Räume durch ein Zusammenspiel der Wirkungen von Hitze und Luftschadstoffen [17]. Anderson et al. [18] konnten in einer Studie, an der 12,5 Mio. ältere Menschen in 213 städtischen Bezirken der USA teilnahmen, bei einem Anstieg der täglichen Umgebungstemperatur um jeweils 10 °Fahrenheit (umgerechnet ca. 5,6 °C) eine um 4,3 % erhöhte Rate an Krankenhauseinweisungen pro Tag aufgrund einer chronisch obstruktiven Lungenerkrankung (COPD) aufzeigen.

Im nationalen Kontext ist die Betrachtung der Morbidität aufgrund von thermischer Belastung im Vergleich zur Mortalität eher unterrepräsentiert. Möglicherweise ist das auf die Datenlage zurückzuführen. Routinedatensätze der Gesetzlichen Krankenversicherung (GKV) bieten aber grundsätzlich die Möglichkeit, Assoziationen von thermischer Belastung und hitzeassoziierten Erkrankungen zu untersuchen [19]. Ziel dieser Untersuchung ist es, anhand von GKV-Routinedaten die Einflüsse klimatischer Variabilität auf die Morbidität am Beispiel hitzeassoziierter Erkrankungen in Deutschland zu analysieren und die Möglichkeiten und Grenzen von GKV-Routinedaten für Fragestellungen dieser Art zu beschreiben.

## Methoden

### Datensatz und Datenvorbereitung

Die vorliegende Studie basiert auf bundesweiten Abrechnungsdaten der Betriebskrankenkasse (BKK – Landesverband NORDWEST KdöR, Essen) der Jahre 2012–2021 für den ambulanten und stationären Sektor. Der vorliegende Datensatz beinhaltet ca. 11,08 Mio. [20] gesetzlich Krankenversicherte in Deutschland, was etwa 15 % der gesamten Bevölkerung in Deutschland entspricht. Der Datensatz umfasst die quartalsweise erfassten Fallzahlen pro 100.000 Versicherte für die folgenden vier Erkrankungsgruppen, die mit Hitze assoziiert und ICD-10-codiert sind: T67 (Schäden durch Hitze und Sonnenlicht), E86 (Volumenmangel), N17 (akutes Nierenversagen) und N19 (Niereninsuffizienz). Die Erkrankungsfälle pro 100.000 Versicherte sind in ambulante und Krankenhausentlassungsdiagnosen (hier auch beschrieben als stationär) unterteilt. Die Auswahl der hier berücksichtigten Diagnosen erfolgte literaturbasiert (z. B. [21–24]). Ergänzend ist anzumerken, dass N17 (akutes Nierenversagen) und N19 (Niereninsuffizienz) hier auch deswegen berücksichtigt wurden, weil beide Diagnosen eine mögliche Folge des Volumenmangels darstellen. Die Daten liegen auf Ebene der Bundesländer vor.

Zur Bestimmung der thermischen Bedingungen wurden die Lufttemperatur, die Wärmebelastung sowie die Sonneneinstrahlung betrachtet, die aus zwei frei verfügbaren Datensätzen stammen. Dabei handelt es sich um Wetterbeobachtungen aus dem Klimadatensatz für Europa (E‑OBS) [25], welcher tägliche Werte auf einem Gitter von ca. 10 × 10 km liefert. Berechnet wurden sowohl für alle Bundesländer als auch für Deutschland das flächengewichtete Mittel der mittleren 2‑Meter-Lufttemperatur, der mittleren Tageshöchsttemperatur (Tmax) sowie die mittlere Anzahl von Hitzetagen (Tmax > 30 °C) und Tropennächten (Tmin > 20 °C) für die jeweiligen Quartale im Zeitraum 2012–2021. Darüber hinaus wurde aus dem **E**uropean-Centre-for-Medium-Range-Weather-Forecasts-(**E**CMWF-)**R**e-**A**nalyse-(ERA-)Datensatz ERA5-HEAT [26] der **U**niversal** T**hermal **C**limate Index (UTCI, [27]) als meteorologischer Indikator für Wärmebelastung entnommen. Die Verwendung der Wärmebelastung anstelle der alleinigen Lufttemperatur ist aus biometeorologischer Sicht sinnvoll, da weitere relevante thermophysiologische Einflussfaktoren wie Luftfeuchte, Windgeschwindigkeit sowie kurz- und langwellige Strahlung berücksichtigt werden. Basierend auf diesen Daten wurden die Flächenmittel der mittleren Tageswerte, der mittleren Tageshöchstwerte sowie der mittleren Hitzetage (UTCI > 32 °C) berechnet. Tab. 1 zeigt eine Übersicht der verwendeten Diagnose- und meteorologischen Daten, inklusive Berechnungsgrundlage und Quelle.

**Tab. 1:** Übersicht der verwendeten Diagnosen und der meteorologischen Daten auf Bundeslandebene, inklusive Quellen

	Variablen	Berechnungsgrundlage	Einheit	Quelle
*ICD-10-Diagnosen*	Schäden durch Hitze und Sonnenlicht (ICD-10 T67)	Ambulante Abrechnungsdaten	Anzahl Fälle pro 100.000 BKK-Versicherte	BKK
		Stationäre Abrechnungsdaten	Anzahl Krankenhausfälle pro 100.000 BKK-Versicherte	
	Volumenmangel (ICD-10 E86)	Ambulante Abrechnungsdaten	Anzahl Fälle pro 100.000 BKK-Versicherte	
		Stationäre Abrechnungsdaten	Anzahl Krankenhausfälle pro 100.000 BKK-Versicherte	
	Niereninsuffizienz (ICD-10 N19)	Ambulante Abrechnungsdaten	Anzahl Fälle pro 100.000 BKK-Versicherte	
		Stationäre Abrechnungsdaten	Anzahl Krankenhausfälle pro 100.000 BKK-Versicherte	
	Akutes Nierenversagen (ICD-10 N17)	Stationäre Abrechnungsdaten	Anzahl Krankenhausfälle pro 100.000 BKK-Versicherte	
*Meteorologie*	Durchschnittstemperatur	Räumlicher Durchschnitt der 2‑Meter-Lufttemperatur	Tagesmittelwerte, °C	E‑OBS
	Mittlere Tageshöchsttemperatur	Räumlicher Durchschnitt der 2‑Meter-Lufttemperatur	Tageshöchstwerte, °C	
	Heiße Tage	Tage mit einer Tageshöchsttemperatur von 30 °C oder höher	Anzahl	
	Tropische Nächte	Tage mit einer Tagesminimumtemperatur von 20 °C oder höher	Anzahl	
	Sonneneinstrahlung	Mittlere kurzwellige Strahlung	Tagesmittelwerte, W/m^2^	
	Heiße Tage – UTCI	Tage mit einem Höchst-UTCI von 32 °C oder höher	Anzahl	ERA5-HEAT
	Maximaler UTCI	Räumlicher Durchschnitt des UTCI	Tageshöchstwerte, °C	
	Mittlerer UTCI	Räumlicher Durchschnitt des UTCI	Tagesmittelwerte, °C	

*BKK* Betriebskrankenkasse, *ICD-10* Internationale statistische Klassifikation der Krankheiten und verwandter Gesundheitsprobleme – Version 10, *UTCI* Universal Thermal Climate Index, *E-OBS* Klimadatensatz für Europa ermittelt aus täglichen Beobachtungsdaten, *ERA5-HEAT* Reanalyse Verfahren zur Erstellung längerfristiger meteorologischer Datensätze

### Deskriptive und räumliche Analysen

Zur Beschreibung der Daten wurden Mittelwert, Minimum und Maximum nach Quartalen (Q; Q2 = April–Juni, Q3 = Juli–September) der Jahre 2012–2021 berechnet. Weiterhin wurden ausgewählte meteorologische Variablen im Jahresverlauf pro Quartal grafisch dargestellt. Die Abbildungen enthalten neben dem Minimum und Maximum auch den Mittelwert Deutschlands sowie den mittleren Wert über alle Jahre hinweg. Mögliche Zusammenhänge zwischen den meteorologischen Variablen und den ausgewählten Diagnosen in Q3 wurden mittels Korrelationsanalyse mit Messwiederholungen [28] untersucht. Dieser Korrelationskoeffizient r_mw_ ermöglicht es, Messwiederholungen zu berücksichtigen, ohne dabei die Annahme der Unabhängigkeit der Daten zu verletzen, die hier aufgrund der mehrmaligen Beobachtungen in jedem Bundesland vorliegen. Um die Unterschiede zwischen den Bundesländern herauszuarbeiten, wurden außerdem Korrelationskoeffizienten r pro Bundesland berechnet. Neben dem Korrelationskoeffizienten r_mw_ werden das zugehörige 95 %-Konfidenzintervall und der *p*-Wert berichtet. Ein *p*-Wert kleiner als 0,05 wurde als statistisch signifikant angesehen. Als Sensitivitätsanalyse wurden diese Zusammenhänge auch für Q2 von 2012–2021 analysiert, um die Robustheit der Ergebnisse zu testen. Die Analysen wurden mit R 4.2.3 (R Core Team 2021, Wien, Österreich) und dem Paket rmcorr [29] durchgeführt.

## Ergebnisse

### Klimatische Entwicklung

**Abb. 1:**
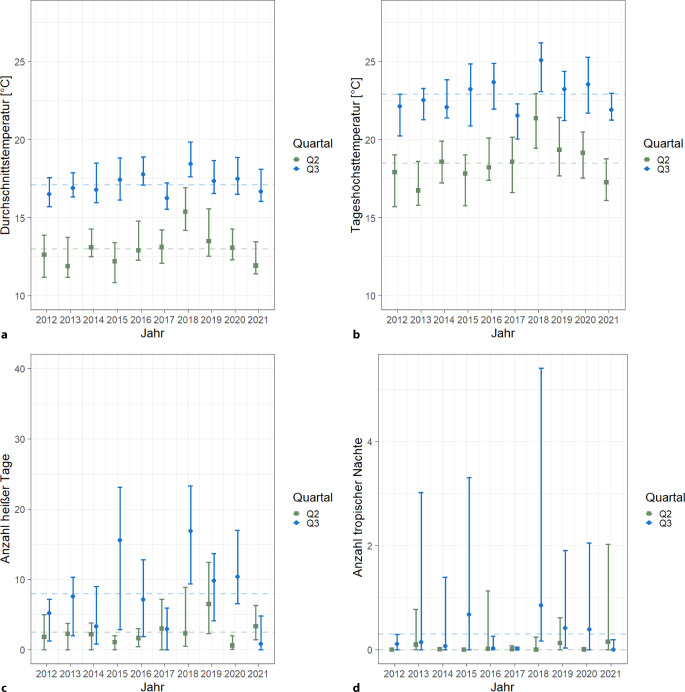
Ausgewählte Klimavariablen für Deutschland im zweiten (Q2) und dritten Quartal (Q3) für die Jahre 2012–2021. Dargestellt sind die Mittelwerte, Maximum und Minimum für die Variablen **a** Durchschnittstemperatur, **b** mittlere Tageshöchsttemperatur, **c** Anzahl heiße Tage und **d** Anzahl Tropennächte basierend auf den Daten für die einzelnen Bundesländer. (Quelle: eigene Abbildung)

Abb. 1 zeigt für ausgewählte Klimavariablen im Überblick, dass bundesweit vor allem die Jahre 2016, 2018 und 2020 hohe Durchschnittstemperaturen und auch hohe Tageshöchsttemperaturen aufwiesen. Heiße Tage waren vor allem in den Jahren 2015, 2018, 2019 und 2020 zu beobachten. Tropische Nächte sind im Vergleich selten und traten eben in diesen Jahren vereinzelt auf. Die entsprechenden Größen der Wärmebelastung haben einen ähnlichen Verlauf, da diese eng mit der Lufttemperatur verbunden sind (nicht gezeigt).

Die mittlere Häufigkeit von Schäden durch Hitze und Sonnenlicht (T67) und Volumenmangel (E86) in den Q2 und Q3 in Deutschland sind in Tab. 2 dargestellt sowie die jährlichen Maximum- und Minimumwerte über den Zeitraum 2012–2021. In Q3 sind die Diagnosen häufiger als in Q2 zu beobachten.

**Tab. 2:** Häufigkeit pro 100.000 Versicherte der hitzeassoziierten Diagnosen und Durchschnittswerte der Flächenmittel der Klimavariablen, in den Quartalen Q2 und Q3 in Deutschland, 2012–2021

ICD-10-Diagnosen	Häufigkeit pro 100.000 Versicherte, Durchschnitt, Minimum und Maximum (Deutschland, 2012–2021)	
	Q2	Q3
Schäden durch Hitze und Sonnenlicht (ICD-10 T67; ambulante Fälle)	41,5 (26,0–61,8)	46,4 (25,3–72,2)
Schäden durch Hitze und Sonnenlicht (ICD-10 T67; stationäre Fälle)	2,3 (0,9–3,3)	3,1 (0,8–7,2)
Volumenmangel (ICD-10 E86; ambulante Fälle)	100,5 (81,2–112,5)	109,0 (88,0–127,4)
Volumenmangel (ICD-10 E86; stationäre Fälle)	346,1 (295,7–380,4)	366,3 (320,7–403,0)
Niereninsuffizienz (ICD-10 N19; ambulante Fälle)	770,5 (713,8–824,4)	775,5 (716,4–817,2)
Niereninsuffizienz (ICD-10 N19; stationäre Fälle)	60,2 (49,3–69,8)	62,8 (51,8–72,7)
Akutes Nierenversagen (ICD-10 N17; ambulante Fälle)	8,3 (5,4–11,7)	8,8 (5,3–12,4)
Akutes Nierenversagen (ICD-10 N17; stationäre Fälle)	38,6 (20,7–48,8)	36,8 (17,1–47,2)
*Klimavariable*	*Durchschnitt, Maximum und Minimum, 2012–2021*	
	Q2	Q3
Durchschnittstemperatur	13,0 °C (11,9–15,4)	17,1 °C (16,2–18,4)
Tageshöchsttemperatur	18,5 °C (16,7–21,4)	22,9 °C (21,5–25,1)
Heiße Tage	2,5 Tage (0,6–6,5)	8,0 Tage (0,8–16,9)
Tropische Nächte	0 Nächte (0–0,2)	0,3 Nächte (0–0,9)
Mittlerer UTCI	10,7 °C (9,0–13,6)	16,0 °C (15,1–17,7)
Maximaler UTCI	19,9 °C (18,0–22,7)	24,3 °C (23,3–26,4)
Heiße Tage basierend auf dem UTCI	6,2 Tage (3,0–10,6)	17,7 Tage (8,4–30,3)
Sonneneinstrahlung	203,4 W/m^2^ (179,3–232,2)	184,0 W/m^2^ (167,7–199,0)

*ICD-10* Internationale statistische Klassifikation der Krankheiten und verwandter Gesundheitsprobleme – Version 10, *UTCI* Universal Thermal Climate Index

### Zusammenhänge zwischen Hitze und Erkrankungen

Aufgrund der geringeren Anzahl stationärer Fälle mit Schäden durch Hitze und Sonnenlicht gegenüber den ambulanten Fällen sowie der geringeren Zahl ambulanter Fälle mit Volumenmangel werden im Folgenden nur die Ergebnisse für die jeweils häufigere Gruppe präsentiert (Tab. 3). Die Diagnosen für Schäden durch Hitze und Sonnenlicht (ambulant; r_mw_ = 0,70 (KI: 0,60–0,77); *p* < 0,001) sowie für Volumenmangel (stationär; r_mw_ = 0,59 (KI: 0,47–0,68); *p* < 0,001) sind für Q3 stark mit erhöhten Durchschnittstemperaturen korreliert, ebenso mit der Anzahl heißer Tage (r_mw_ = 0,86 (KI: 0,81–0,90), *p* < 0,001 und r_mw_ = 0,50 (KI: 0,37–0,61); *p* < 0,001). Die Korrelationen beider Erkrankungen mit dem UTCI und damit mit dem thermischen Wohlbefinden fallen jedoch geringer aus als die Koeffizienten mit den entsprechenden Lufttemperaturgrößen. Die Korrelationen mit der Sonneneinstrahlung fallen ebenfalls geringer aus, sind aber statistisch signifikant (Tab. 3; *p* < 0,001).

**Tab. 3:** Korrelationen zwischen hitzeassoziierten Diagnosen und Klimavariablen für die Quartale Q2 und Q3 im Zeitraum 2012–2021 in Deutschland

ICD-10-Diagnosen	Klimavariable	Korrelationskoeffizient r_mw_ und 95 %-Konfidenzintervall	
		Q2	Q3
Schäden durch Hitze und Sonnenlicht (ICD-10 T67; ambulant)	Durchschnittstemperatur	0,43 [0,29; 0,56] ***	0,70 [0,60; 0,77] ***
	Tageshöchsttemperatur	0,40 [0,26; 0,53] ***	0,72 [0,63; 0,79] ***
	Heiße Tage	0,76 [0,68; 0,82] ***	0,86 [0,81; 0,90] ***
	Tropische Nächte	0,25 [0,09; 0,39] **	0,60 [0,49; 0,70] ***
	Mittlerer UTCI	0,29 [0,13; 0,43] ***	0,51 [0,37; 0,62] ***
	Maximaler UTCI	0,27 [0,11; 0,41] **	0,53 [0,41; 0,65] ***
	Heiße Tage basierend auf dem UTCI	0,57 [0,45; 0,67] ***	0,66 [0,56; 0,75] ***
	Sonneneinstrahlung	0,11 [−0,06; 0,27]	0,53 [0,40; 0,64] ***
Volumenmangel (ICD-10 E86; stationär)	Durchschnittstemperatur	0,25 [0,09; 0,40] **	0,59 [0,47; 0,68] ***
	Tageshöchsttemperatur	0,23 [0,07; 0,38] **	0,56 [0,44; 0,66] ***
	Heiße Tage	0,40 [0,25; 0,53] ***	0,50 [0,37; 0,61] ***
	Tropische Nächte	0,11 [−0,05; 0,27]	0,33 [0,17; 0,47] ***
	Mittlerer UTCI	0,12 [−0,05; 0,27]	0,47 [0,33; 0,58] ***
	Maximaler UTCI	0,10 [−0,06; 0,26]	0,46 [0,32; 0,58] ***
	Heiße Tage basierend auf dem UTCI	0,28 [0,12; 0,42] ***	0,38 [0,23; 0,51] ***
	Sonneneinstrahlung	0,01 [−0,15; 0,17]	0,36 [0,21; 0,49] ***
Niereninsuffizienz (ICD-10 N19; ambulant)	Durchschnittstemperatur	0,24 [0,08; 0,39]	0,36 [0,21; 0,49]
	Tageshöchsttemperatur	0,28 [0,12; 0,42]	0,31 [0,15; 0,45]
	Heiße Tage	0,17 [0,01; 0,32]	0,21 [0,04; 0,36]
	Tropische Nächte	0,07 [−0,09; 0,23]	0,10 [−0,07; 0,26]
	Mittlerer UTCI	0,21 [0,05; 0,36]	0,29 [0,13; 0,43]
	Maximaler UTCI	0,21 [0,04; 0,36]	0,25 [0,09; 0,40]
	Heiße Tage basierend auf dem UTCI	0,06 [−0,10; 0,22]	0,09 [−0,08; 0,25]
	Sonneneinstrahlung	0,22 [0,06; 0,37]	0,06 [−0,11; 0,22]
Akutes Nierenversagen (ICD-10 N17; stationär)	Durchschnittstemperatur	−0,04 [−0,20; 0,12]	0,06 [−0,10; 0,22]
	Tageshöchsttemperatur	−0,11 [−0,27; 0,05]	0,09 [−0,08; 0,24]
	Heiße Tage	−0,26 [−0,41; −0,10]	0,17 [0,01; 0,33]
	Tropische Nächte	−0,25 [−0,40; −0,09]	0,05 [−0,12; 0,21]
	Mittlerer UTCI	0,05 [−0,11; 0,21]	−0,01 [−0,17; 0,15]
	Maximaler UTCI	−0,04 [−0,20; 0,13]	0,01 [−0,15; 0,17]
	Heiße Tage basierend auf dem UTCI	0,03 [−0,13; 0,19]	0,22 [0,06; 0,37]
	Sonneneinstrahlung	−0,54 [−0,65; −0,42]***	−0,09 [−0,25; 0,07]

Signifikanzen: * *p* < 0,05; ** *p* < 0,01; *** *p* < 0,001

*ICD-10* Internationale statistische Klassifikation der Krankheiten und verwandter Gesundheitsprobleme – Version 10, *UTCI* Universal Thermal Climate Index

Die anderen beiden untersuchten Erkrankungen, Niereninsuffizienz (ambulant) und akutes Nierenversagen (stationär), zeigen bei bundesweiter Betrachtung keine Korrelationen mit der Lufttemperatur und der Wärmebelastung.

### Sensitivitätsanalyse

Entsprechend der Jahreszeit ist die Anzahl der heißen Tage in Q2 deutlich geringer als in Q3. Da jedoch auch im Frühjahr/Frühsommer hohe Temperaturen erreicht werden können und zudem der Körper physiologisch noch nicht ausreichend gegenüber Hitze adaptiert ist, kann auch hier von einer gesundheitlichen Belastung ausgegangen werden. Dies zeigt sich auch in den Analysen, denn auch in Q2 ist ein Anstieg von Schäden durch Hitze und Sonnenlicht bei häufigeren heißen Tagen (r_mw_ = 0,76 (KI: 0,68–0,82); *p* < 0,001) und heißen Tagen basierend auf dem UTCI (r_mw_ = 0,66 (KI: 0,56–0,75); *p* < 0,001) zu beobachten. Der Volumenmangel ist in Q2 deutlich mit heißen Tagen (r_mw_ = 0,40 (KI: 0,25–0,53); *p* < 0,001) und nur schwach mit der Durchschnittstemperatur (r_mw_ = 0,25 (KI: 0,09–0,39); *p* = 0,002) sowie mit der Anzahl heißer Tage basierend auf dem UTCI (r_mw_ = 0,28 (KI: 0,12–0,42); *p* < 0,001) korreliert. Für die Diagnose Niereninsuffizienz und akutes Nierenversagen waren bundesweit auch in Q2 keine signifikanten Korrelationen zu beobachten. In Q2 ist die Niereninsuffizienz nur in einzelnen Bundesländern positiv mit der Durchschnittstemperatur (Sachsen-Anhalt, *r* = 0,66 (KI: 0,06–0,91); *p* = 0,036), Tageshöchsttemperatur (Sachsen-Anhalt, *r* = 0,69 (KI: 0,10–0,92), *p* = 0,028) oder mit heißen Tagen basierend auf dem UTCI (Saarland, *r* = 0,70 (KI: 0,13–0,92); *p* = 0,023) korreliert.

### Räumliche Variationen

**Abb. 2:**
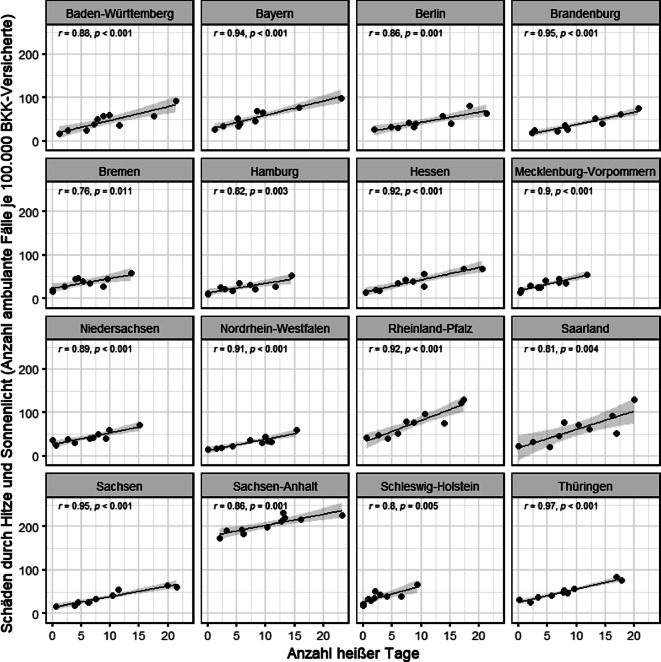
Heiße Tage und Häufigkeit von Schäden durch Hitze und Sonnenlicht (ICD-10 T67; ambulante ICD-10-Diagnosen) pro 100.000 Versicherte pro Jahr (2012–2021) in Q3 in den einzelnen Bundesländern. *ICD-10* Internationale statistische Klassifikation der Krankheiten und verwandter Gesundheitsprobleme – Version 10. (Quelle: eigene Abbildung)

Wie in den Abb. 2 und 3 zu sehen ist, können die Korrelationen für einzelne Bundesländer höher ausfallen als im Bundesdurchschnitt. Die räumlichen Variationen der Häufigkeiten hitzeassoziierter Erkrankungen einerseits als auch der Zusammenhänge zwischen diesen Erkrankungen und der Lufttemperatur in den einzelnen Bundesländern sind sehr groß. Alle Bundesländer weisen einen klaren Zusammenhang zwischen der Anzahl heißer Tage und Schäden durch Hitze und Sonne in Q3 auf (Abb. 2).

**Abb. 3:**
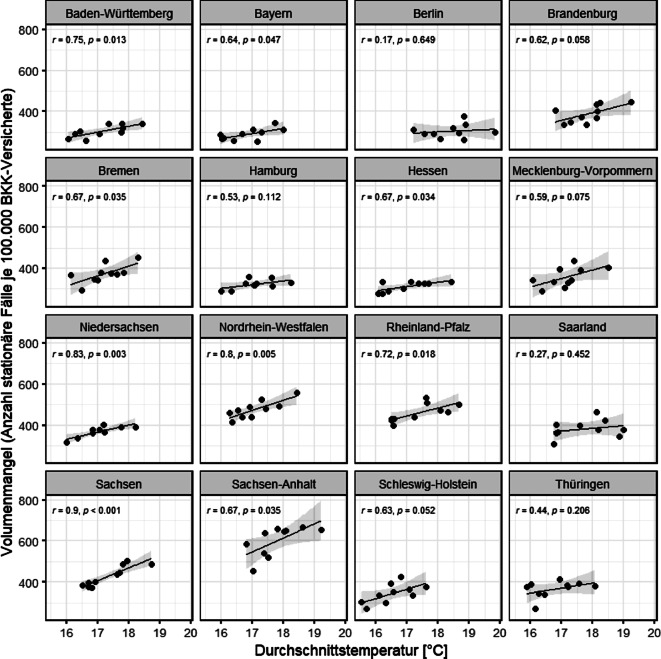
Durchschnittstemperatur und Häufigkeit von Volumenmangel (ICD-10 E86; stationäre ICD-10-Diagnosen) pro 100.000 Versicherte pro Jahr (2012–2021) in Q3 in den einzelnen Bundesländern. *ICD-10* Internationale statistische Klassifikation der Krankheiten und verwandter Gesundheitsprobleme – Version 10. (Quelle: eigene Abbildung)

Der Volumenmangel weist in 9 von 16 Bundesländern einen signifikanten Zusammenhang mit der Durchschnittstemperatur in Q3 (Abb. 3) auf, jedoch nicht in Berlin, Brandenburg, Hamburg, Saarland, Schleswig-Holstein, Mecklenburg-Vorpommern und Thüringen.

## Diskussion

Das übergeordnete Ziel dieser Analyse war die Untersuchung der Einflüsse klimatischer Variabilität auf die Morbidität am Beispiel hitzeassoziierter Erkrankungen in Deutschland anhand von GKV-Routinedaten. Die Ergebnisse dieser Studie haben dabei teilweise deutliche korrelative Zusammenhänge zwischen dem Auftreten von hoher thermischer Belastung und ausgewählten hitzeassoziierten Erkrankungen für den ambulanten und stationären Sektor aufgezeigt, die den Ergebnissen anderer Studien [30] entsprechen. Zudem hat sich gezeigt, dass sich der Jahresgang (hier in Q2 und Q3) der Temperatur grundsätzlich auch in den Diagnosehäufigkeiten wiederfindet. Dies betrifft vor allem die Schäden durch Hitze und Sonnenlicht (ICD-10 T67) sowie Volumenmangel (ICD-10 E86). Je nach Quartal, ambulant oder stationär und Diagnose waren die Assoziationen mehr oder weniger deutlich ausgeprägt. Sowohl in Q2 als auch in Q3 sowie bei ambulanten und stationären Fällen war die Diagnosehäufigkeit bei ICD-10 T67 und ICD-10 E86 in Sachsen-Anhalt besonders auffallend. Da sich Sachsen-Anhalt aus klimatologischer, bevölkerungs- und gesundheitsspezifischer Sicht nicht grundlegend von den umliegenden Bundesländern unterscheidet, ist hier aber eventuell von einem Artefakt auszugehen. Anzumerken ist, dass möglicherweise eine Unterschätzung der Fallzahlen, vor allem bei ICD-10 T67 (Schäden durch Hitze und Sonnenlicht), vorliegt, da diese vermutlich seitens der Ärzt:innen selten als Zusatzdiagnose angegeben wird.

Bei Niereninsuffizienz (ICD-10 N19) und akutem Nierenversagen (ICD-10 N17) hat sich kein eindeutiger Zusammenhang zu thermischen Bedingungen gezeigt. Niereninsuffizienz war nur in einzelnen Fällen (Bundesland Brandenburg, r = 0,57 (KI: −0,09–0,09); *p* = 0,086) im Juli–September (Q3) mit der Durchschnittstemperatur korreliert. Zudem zeigten sich sowohl für den ambulanten als auch für den stationären Bereich nahezu gleich hohe Fallzahlen für Nierenerkrankungen in Q2 und Q3 bei höheren Durchschnittstemperaturen in Q3. Dies deutet darauf hin, dass keine ausgeprägte Assoziation zwischen Hitze und Niereninsuffizienz (ICD-10 N19) sowie Nierenversagen (ICD-10 N17) besteht bzw. dies aus den GKV-Daten nicht hervorgeht.

Ein Vergleich der Ergebnisse dieser Studie mit anderer Studien ist schwierig, da Morbidität in der Regel im Kontext anderer Diagnosen, z. B. kardiovaskulare Diagnosen [31] oder kardiorespiratorische Diagnosen [32], und vor allem aufgrund einer Einweisung in Notfallaufnahmen betrachtet wurde. In einem Review konnten Faurie et al. [24] mit gepoolten Ergebnissen zeigen, dass ein Temperaturanstieg um 1 °C mit einem 18 %igen Anstieg der hitzebedingten Krankheitsmorbidität (hier definiert mit ICD-10 T67, E86, X30) assoziiert (RR 1,18, 95 % KI 1,16–1,19) ist. Dieser Effekt war für Hitzeerkrankungen oder Hitzschlag (RR 1,45, 95 % KI: 1,38–1,53) größer als bei Volumenmangel (RR 1,02, 95 % KI: 1,02–1,03). Basu et al. [33] haben zwischen 2005 und 2008 die Assoziationen zwischen Temperatur und Notfallaufnahmen in Kalifornien untersucht. Dabei zeigte sich unter anderem bei erhöhter Temperatur (zusätzliches Risiko pro 10 °Fahrenheit (entspricht umgerechnet ca. 5,6 °C) in %) am selben Tag eine Zunahme der Notaufnahmen mit Dehydrierung (25,6 % (KI: 21,9–29,4)), akutem Nierenversagen (15,9 % (KI: 12,7–19,3)) und Hitzeerkrankungen (393,3 % (KI: 331,2–464,5)). Das Risiko variierte häufig je nach Alter oder ethnischer Gruppe.

Schwarz et al. [34] haben die hitzeassoziierten Krankenhauseinweisungen zwischen 1999 und 2012 in Südkalifornien analysiert, und zwar außerhalb der Sommersaison. Es zeigte sich, dass auch im Herbst und im Frühjahr das Risiko einer Krankenhauseinweisung für Dehydrierung (OR: 1,23, 95 % KI: 1,04, 1,45 bzw. OR: 1,47 95 % KI: 1,25, 1,71) und akutes Nierenversagen (OR: 1,35, 95 % KI: 1,15, 1,58 bzw. OR: 1,39, 95 % KI: 1,19, 1,63) während eintägiger extremer Hitzeereignisse anstieg. Die Ergebnisse sind insofern von Bedeutung, da Auswirkungen von Hitze auch im Frühjahr relevant sein können. Dies hat sich in dieser Studie u. a. auch in der Assoziation von Schäden durch Hitze und Sonnenlicht (ICD-10 T67) mit heißen Tagen in Q2 angedeutet. Letztere können vereinzelt auch schon im Mai und Juni auftreten, das heißt auch dann, wenn die thermophysiologische Anpassung des Körpers gegenüber hohen Temperaturen noch gering ist.

Die Analyse hitzeassoziierter Erkrankungen mittels Abrechnungsdaten hat gezeigt, dass GKV-Routinedaten durchaus sehr deutliche Effekte von thermisch auffälligen Zeiträumen zeigen und für die Analyse der temperaturbedingten Morbidität wie Hitze- und Sonnenschäden und Volumenmangel grundsätzlich verwendet werden können. GKV-Routinedaten haben vor allem den Vorteil, dass Erkrankungen in der Bevölkerung flächendeckend von erheblich mehr Versicherten regelmäßig erfasst als beispielsweise in Kohortenstudien eingeschlossen werden können. Zudem kann sich die breite räumliche Verteilung von Erkrankungshäufigkeiten besser für die Analyse der räumlich differenzierten Auswirkungen hoher thermischer Belastungen eignen, wenn zum Beispiel Unterschiede zwischen städtischen und ländlichen Umgebungen gezeigt werden. Darüber hinaus bieten GKV-Routinedaten die Möglichkeit, Morbiditäten, die außerhalb der stationären Versorgung auftreten, im Zusammenhang mit hohen thermischen Belastungen zu untersuchen, was bislang vernachlässigt wurde, obwohl die Ereignisrate zum Beispiel gegenüber hitzeassoziierten Krankenhauseinweisungen um ein Vielfaches höher liegt [35] und daher aus Public-Health- und gesundheitsökonomischer Perspektive von hoher Relevanz ist.

Wenngleich es zahlreiche weitere internationale epidemiologische Studien zur Wirkung von Hitze auf Morbidität und vor allem Mortalität gibt, bleibt eine Quantifizierung im Kontext klimatischer Veränderungen und ihrer Auswirkungen auf die Gesundheit jedoch sehr komplex und kann bislang nur unvollständig bemessen werden. Dies ist vermutlich auf die Datenlage, insbesondere im Bereich der Gesundheitsdaten, zurückzuführen. Die Weltorganisation für Meteorologie (WMO) definiert „Klimawandel“ mit Veränderungen über einen Zeitraum von 30 Jahren [36]. Bei einem besonders heißen Sommer mit hoher Mortalität könnte es sich demnach, losgelöst vom Klimawandel, um ein singuläres Ereignis handeln. Es ist also die Betrachtung langer Zeiträume notwendig, um den Einfluss klimatischer Veränderungen abbilden zu können. In der Regel existieren Gesundheitsdaten (z. B. GKV-Routinedaten, Kohortendaten, Registerdaten) nicht über so lange Zeiträume, was unter Umständen auf den Datenschutz zurückzuführen ist. So regelt das Sozialgesetzbuch (SGB) Fünftes Buch (V) (§ 304 Abs. 1), dass Krankenkassen ihre Daten spätestens nach 10 Jahren löschen müssen [37], ein Zeitraum, der Aussagen zu den Folgen des Klimawandels anhand dieser Daten kaum zulässt. Der in dieser Studie begrenzte Zeitraum von 10 Jahren ist Grund dafür, warum hier auf eine Trendanalyse verzichtet wurde.

Darüber hinaus ist aufgrund der hohen raumzeitlichen Variabilität von Hitzeereignissen auch in den Gesundheitsdaten eine entsprechende hohe räumliche und zeitliche Auflösung notwendig, die oftmals nicht gewährleistet werden kann, da bei einer sehr kleinräumigen Betrachtung beispielsweise die erforderliche Mindestfallzahl nicht mehr erreicht werden kann. Eine ähnliche Limitation ergibt sich bei der zeitlichen Auflösung, da bei pauschalierter Abrechnung ambulanter ärztlicher Leistungen kein taggenauer Leistungsbezug hergestellt werden kann. Ambulante Diagnosen liegen in der Regel auf Quartalsebene vor [19], sodass ein genauer Zusammenhang zu Hitzewellen, die in der Regel nur einige Tage andauern, nicht erfasst werden kann. Dieser Aspekt erschwert zudem die Vergleichbarkeit mit anderen Studien, da diese in der Regel die Kurzzeiteffekte (z. B. Notfallaufnahmen) untersuchen, jedoch keine Aussagen zu Langzeiteffekten machen. Die grobe Auflösung der Gesundheitsdaten lässt auch eine Untersuchung von kleinräumiger Variation der meteorologischen Größen wie innerstädtische Temperaturunterschiede nicht zu, obwohl in der Regel langjährige meteorologische Daten bzw. Klimadaten in zeitlich und räumlich hochaufgelöster Form vorliegen (z. B. [38]).

Wenngleich sich in den hier generierten Ergebnissen deutliche Assoziationen zwischen den meteorologischen Parametern und medizinischen Diagnosen zeigen, stellt sich die Frage nach der Kausalität der Ergebnisse. Dazu ist festzuhalten, dass die Betrachtung eines „nur“ 10-jährigen Zeitraums keine direkten, kausalen Schlussfolgerungen über die Auswirkungen des Klimawandels auf die Veränderung der Häufigkeit der hier berücksichtigten Diagnosen erlaubt, da der Wechsel von wärmeren und kälteren Jahren bzw. Sommern zum größten Teil auf natürliche Schwankungen in diesem Zeitraum zurückzuführen ist. Es ist jedoch noch ein weiterer Aspekt von Bedeutung, und zwar die Berücksichtigung zusätzlich wirkender gesundheitsbeeinflussender Faktoren, wie Health Literacy, Lebensstil, soziale Lage, Altersstruktur der Bevölkerung, Systemveränderungen oder auch Anpassungsmaßnahmen, die sich parallel zu den klimatischen Veränderungen auch verändern. Das gilt nicht nur für eine retrospektive Betrachtung, sondern insbesondere auch für die Projektion zukünftiger klimabedingter Gesundheitsrisiken [39].

Die meisten Bewertungen zukünftiger hitzebedingter Gesundheitsrisiken beruhen auf der Projektion von Hitzeschäden, die den soziökonomischen Bedingungen überlagert werden, d. h. andere Einflussgrößen vernachlässigen [40]. Solch eine Vereinfachung komplexer Zusammenhänge, wie die Annahme, dass ein einziger Temperaturwert Mortalität adäquat bestimmt, oder die Verwendung ungeeigneter Hitzestressmetriken, kann zu unrealistischen Projektionen des Spektrums künftiger hitzebedingter Gesundheitsfolgen führen, von Wohlbefinden über Krankheit bis hin zum Tod. Die Nichtberücksichtigung der menschlichen Anpassungsfähigkeit führt zudem zu weiteren Unwägbarkeiten [41].

Darüber hinaus kommt erschwerend hinzu, dass die gesundheitsbeeinflussenden Faktoren nicht alleine, sondern in Kombination (z. B. Temperatur und bodennahes Ozon) wirken und sich ihr negativer Einfluss damit nochmals verstärken kann [42]. Mit Blick auf die Berücksichtigung soziodemografischer Einflüsse (z. B. als Confounder) hat sich zudem gezeigt, dass das Problem geeigneter Daten nicht nur das Gesundheitswesen betrifft, sondern zumindest in Teilen auch andere Themenbereiche (Sozialstruktur etc.). Zwar liegen auf Bevölkerungsebene Daten zur sozialen Lage nach Bundesländern vor, jedoch lassen sich diese Daten nicht einfach mit den Versichertendaten korrelieren bzw. auf die Versichertenpopulation beziehen. Eine Möglichkeit der Annäherung bestünde darin, aus den Stammdaten der GKV den Tätigkeitsschlüssel heranzuziehen, aus dem unter anderem Angaben zum beruflichen Bildungsabschluss von Versicherten hervorgehen. Aus dem Versichertenstatus (Pflichtmitgliedschaft, freiwillige Mitgliedschaft oder Familienversicherung) können zudem zumindest grobe Aussagen zum Einkommen abgeleitet werden. Im Rahmen einer sich bereits in Arbeit befindlichen Folgestudie werden neben weiteren Diagnosen auch soziodemografische Angaben berücksichtigt.

Insgesamt lässt sich die Forderung ableiten, die Datengrundlage zur Analyse der gesundheitlichen Folgen des Klimawandels zu verbessern und den Zugang zu erleichtern. Dies erfordert in erster Linie ein Umdenken dahin gehend, die existierenden Daten auch für die Wissenschaft und Analysezwecke nutzbar zu machen, das heißt ausreichend lange Zeiträume sowie eine hohe räumliche- und zeitliche Auflösung der Daten für die Forschung bereitzustellen.

Die Klimaausblicke für Deutschland [43] zeigen, dass die Durchschnittstemperatur und auch die Anzahl der heißen Tage zunehmen werden und somit eine entsprechend weiter zunehmende Gesundheitsbelastung durch den Klimawandel erwartet werden muss. Die Analyse dieser hochkomplexen Zusammenhänge erfordert eine gemeinsame Betrachtung von Umwelt-, ökologischen und sozialen Aspekten [44] sowie eine Intensivierung der Zusammenarbeit von Klima‑, Sozial- und Gesundheitswissenschaften [41, 42].

## Fazit

Dieser Beitrag untersucht die Folgen hoher thermischer Belastungen auf Grundlage von GKV-Routinedaten zwischen 2012–2021 und ist damit nach unserem Wissen der erste seiner Art in Deutschland. Damit kann er in erster Linie als Basis für weitere GKV-Analysen zu den Folgen klimatischer Veränderungen auf die Gesundheit angesehen werden. Wenngleich die Anforderungen an Daten zur Untersuchung der Folgen des Klimawandels auf die Gesundheit hoch sind, hat diese Auswertung verdeutlicht, dass sich auch in GKV-Daten Hinweise auf mögliche Folgen klimatischer Veränderungen auf die Gesundheit finden lassen. So konnten deutliche Assoziationen zwischen ausgewählten Diagnosen (z. B. Schäden durch Hitze und Sonnenlicht, T67) und meteorologischen Variablen (z. B. Anzahl heißer Tage) identifiziert werden. Aus dieser Untersuchung lässt sich jedoch auch die Erkenntnis gewinnen, dass eine Verbesserung der Datenlage, etwa durch längere Zeitreihen sowie eine höhere raumzeitliche Auflösung von Gesundheitsdaten, zur Beantwortung von Fragen zu den Folgen des Klimawandels auf die Gesundheit notwendig ist und angestrebt werden sollte.
